# Editorial: Preventing cardiovascular complications of type 2 diabetes, volume II

**DOI:** 10.3389/fendo.2026.1792059

**Published:** 2026-01-29

**Authors:** Nadia Di Pietrantonio, Maria Pompea Antonia Baldassarre, Ilaria Cappellacci, Teresa Paolucci, Kyoungmin Park, Caterina Pipino

**Affiliations:** 1 Department of Medical, Oral and Biotechnological Sciences, G. d’Annunzio University, Chieti, Italy; 2 Center for Advanced Studies and Technology (CAST), G. d’Annunzio University, Chieti, Italy; 3 Department of Medicine and Aging Sciences, G. d’Annunzio University, Chieti, Italy; 4 Department of Medical, Oral and Biotechnological Sciences, Center of Rehabilitation, Sports and Disability (CARES), G. d’Annunzio University, Chieti, Italy; 5 BIND Center, Physical and Rehabilitation Medicine, G. d’Annunzio University, Chieti, Italy; 6 Research Division, Joslin Diabetes Center, Department of Medicine, Harvard Medical School, Boston, MA, United States

**Keywords:** cardiovascular risk, glycemic variability, novel biomarkers, precision medicine, type 2 diabetes

Diabetes mellitus represents a major global health challenge, with a rapidly increasing prevalence worldwide, and is associated with a markedly elevated risk of adverse health outcomes, particularly cardiovascular disease (CVD) (1). Individuals with diabetes have a substantially higher likelihood of developing vascular complications, which are classified as microvascular—such as diabetic nephropathy and retinopathy—or macrovascular, including coronary artery disease, stroke, and peripheral artery disease. Consequently, substantial efforts are required to implement innovative technologies and strategies aimed at preventing cardiovascular complications in individuals with type 2 diabetes (T2D), through both lifestyle modification and early, accurate, and effective diagnosis.

## Unveiling new risk markers for cardiovascular outcomes in diabetes

The search for new strategies to prevent diabetes and its cardiovascular complications increasingly emphasizes the identification of novel biomarkers and the re-evaluation of established ones, aiming to enable earlier and more precise risk assessment (2). One such area of interest is glycemic variability.

Wu et al. conducted a systematic review and meta-analysis to determine whether fluctuations in glycated hemoglobin (HbA1c) predict cardiovascular risk in patients with T2D. While mean HbA1c remains the standard measure of long-term glycemic control, it fails to capture short-term glucose swings and can be influenced by factors such as anemia or pregnancy. Evidence indicates that glycemic variability-measured through HbA1c standard deviation, coefficient of variation, and indices like the HbA1c variability score or hemoglobin glycation index—may independently contribute to vascular outcomes. Wu et al. provide the first population-level evidence that HbA1c fluctuations themselves significantly increase cardiovascular risk, even when mean HbA1c levels are within target ranges, highlighting a new avenue for precision glycemic management.

Beyond glycemic variability, lipid-based markers are emerging as important predictors of both diabetes and cardiovascular complications. The remnant cholesterol to HDL-C ratio (RC/HDL-C) has recently gained attention as an indicator of metabolic disturbances. In a cohort of 7,655 Chinese adults aged ≥60 years, Tang et al. observed that elevated RC/HDL-C levels were independently associated with an increased risk of T2D. The results suggest that RC/HDL-C could enhance existing risk models. Zhang et al. similarly confirmed these findings, demonstrating that participants in the highest RC/HDL-C quartile had the greatest incidence of T2D, reinforcing its role as an independent risk factor in older adults and a potential tool for improving risk stratification in predictive models.

Another readily available biomarker is the Triglyceride–Glucose (TyG) index, a low-cost surrogate marker of insulin resistance. The TyG index appears to reflect aspects of dynamic glycemic instability that may contribute to vascular injury beyond chronic hyperglycemia. In a retrospective analysis of NHANES participants aged ≥60 years, S. Yang et al. found that both very low and very high TyG values were linked to elevated cardiovascular risk, highlighting the complexity of metabolic regulation in older individuals.

Collectively, these studies underscore the critical importance of dynamic metabolic markers in improving risk prediction for diabetes and its cardiovascular complications. Integrating these novel indicators with traditional measures could enable more individualized, precise strategies for early detection, prevention, and management of vascular risk in older adults with or at risk for T2D.

## Integrating metabolomics to uncover cardiovascular risk diabetes

In recent years, cardiovascular phenotyping has advanced through the use of unbiased, high-throughput omics technologies capable of simultaneously analyzing genes, transcripts, proteins, and metabolites (3). These approaches have greatly expanded our understanding of disease pathways and potential biomarkers. In T2D and its cardiovascular complications, integrating multiple omics layers offers the opportunity to detect early endothelial dysfunction, clarify pathophysiological mechanisms, and identify novel predictive markers. Among these technologies, metabolomics is particularly valuable: it reflects both genetic and environmental influences such as diet, lifestyle, medication, microbiome. This makes it a promising tool for developing diagnostic, prognostic, and therapy-response biomarkers, ultimately supporting more personalized diabetes care.

Despite substantial progress, metabolomic interactions between T2D and coronary heart disease (CHD) remain poorly characterized in Middle Eastern populations, where T2D prevalence is among the highest globally. Addressing this gap, Elshrif et al. conducted the first large-scale metabolomics study of T2D and CHD in Qatar. Through univariate and multivariate analyses, pathway enrichment, machine learning, and metabolite-based risk scores, the authors identified marked metabolic differences between T2D patients with and without CHD. Metabolite risk scores showed strong predictive performance, underscoring its potential clinical practicality.

Overall, this work highlights distinct metabolic signatures associated with CHD in T2D patients and proposes candidate biomarkers for earlier detection, improved risk stratification, and future therapeutic targeting in an underrepresented population.

## Natural compounds for preventing diabetic vascular dysfunction

Despite significant advances in diabetes care, lifestyle measures like healthy eating and regular exercise remain the foundation of managing T2D. However, these strategies alone are often not enough to prevent or reduce the vascular complications associated with diabetes, highlighting the need for additional therapeutic approaches. In this context, natural bioactive compounds, especially polyphenols, have attracted growing interest due to their strong anti-inflammatory, antioxidant, and heart-protective effects (4). Olive leaves, in particular, are a largely underutilized source of polyphenols, notably oleuropein, which can reduce oxidative stress, prevent blood clotting, and improve insulin sensitivity and blood sugar control.

The study by Cappellacci et al. observed that extracts from two spanish olive monocultivars, Picual and Changlot Real, reduce endothelial inflammation—a key factor in diabetes-related vascular problems. Using the *in vitro* model of human umbilical vein endothelial cells from women with gestational diabetes, the researchers showed that both extracts could influence the Nuclear Factor kappa-light-chain-enhancer of activated B cells (NF-κB)– Vascular cell adhesion protein 1 (VCAM-1) pathway, lowering inflammation and monocyte adhesion. These results suggest that olive leaf polyphenols may help protect against vascular complications in diabetes, highlight the potential of natural compounds, such as olive-derived polyphenols, to complement existing diabetes management strategies.

## Protecting the diabetic heart: early risk to prevention

T2D significantly increases the risk of CVD by damaging the vascular endothelium and promoting processes that accelerate atherosclerosis and arterial stiffening. These changes contribute directly to the development of myocardial infarction, heart failure, and arrhythmias such as atrial fibrillation, making cardiovascular complications the leading cause of morbidity and mortality in people with diabetes. This underscores the urgent need for early detection of individuals at high cardiac risk and for strategies aimed at protecting heart health in the context of T2D.

Several recent studies have explored practical, non-invasive markers to improve early risk stratification in T2D. Zhao et al. investigated the Glycemia Risk Index (GRI) in relation to carotid intima–media thickness (CIMT), a recognized indicator of subclinical atherosclerosis. In a cohort of 450 hospitalized adults with T2D, the study demonstrated that GRI correlates with CIMT, suggesting its potential as a convenient tool for detecting early macrovascular risk and complementing continuous glucose monitoring-based assessments.

Arterial stiffness represents another early and independent predictor of cardiovascular and renal outcomes. Using brachial–ankle pulse wave velocity (baPWV), He et al. examined its association with the blood urea nitrogen–to–albumin ratio (BAR) in 510 newly diagnosed, drug-naïve T2D patients. The study found a significant positive relationship, indicating that BAR may serve as a simple biomarker to identify early vascular changes in this population.

Insulin sensitivity also plays a key role in cardiovascular risk. Tan et al. evaluated the estimated glucose disposal rate (eGDR) in over 31,000 adults with diabetes from the UK Biobank. Higher eGDR was associated with substantially lower risks of atrial fibrillation, heart failure, and cardiovascular mortality, regardless of genetic risk. These findings position eGDR as a robust and clinically meaningful marker for predicting cardiovascular outcomes in diabetes.

Finally, myocardial fibrosis following acute myocardial infarction (AMI) is a critical determinant of prognosis but is difficult to assess directly during the acute phase. Chen et al. validated a synthetic extracellular volume (ECV) measure derived from cardiac magnetic resonance imaging, which does not require blood sampling. In patients with T2D and AMI, synthetic ECV closely matched conventional ECV measurements and independently predicted major adverse cardiovascular events, demonstrating its potential as a non-invasive prognostic tool.

Taken together, these studies highlight the critical need for early cardiovascular risk detection in diabetes. By incorporating novel heart-focused biomarkers—spanning glycemic instability, arterial stiffness, insulin sensitivity, and imaging markers of myocardial fibrosis—clinicians can more accurately identify individuals at heightened cardiac risk. This enables earlier, targeted interventions aimed at preventing the progression of diabetic heart disease.

## Medical advances in the management of diabetes and vascular disease

Effective clinical management, particularly adequate glycemic control, is essential for preventing cardiovascular complications in diabetes (5). In 2015 when major trials showed that Glucagon Like Peptide-1 receptor agonists (GLP-1RAs) and Sodium/Glucose Transporter 2 (SGLT2) inhibitors significantly reduce cardiovascular and renal events. Along with ACEIs/ARBs, statins, and aspirin, these agents are now strongly recommended for cardiovascular risk reduction in T2D with established or high Atherosclerotic CVD risk.

Despite guideline support, the use of these therapies remains limited. In a large population-based cohort from Xiamen Humanity Hospital (2018–2023), Tian et al. found that patients using evidence-based cardioprotective therapies had lower risk of MACE, heart failure hospitalization, and a reduction in progression to advanced kidney disease compared with non-users.

Aristizábal-Colorado et al. emphasized individualized therapy selection: SGLT2 inhibitors are particularly beneficial in patients with heart or kidney disease, while GLP-1RAs may be preferable for obese individuals or those at high cardiometabolic risk. Importantly, combining both drug classes can offer additive cardiovascular and renal protection.

H. Gao et al. analyzed 14 trials in patients with T2D and heart failure, comparing the effects of different glucose-lowering drugs. Semaglutide showed the strongest overall cardiac benefit by reducing B-type natriuretic peptide. Licogliflozin caused the greatest weight loss, glimepiride reduced glycated hemoglobin the most, and dapagliflozin most improved left ventricular ejection fraction. Safety varied across drugs, with licogliflozin and ipragliflozin showing fewer side effects. Overall, semaglutide emerged as the most comprehensive cardiometabolic option.

Together, these findings reinforce the growing evidence that targeted glucose-lowering therapies, when appropriately selected and effectively implemented, have the potential modify cardiovascular outcomes in people with T2D.

## Conclusion: A wake-up call for cardiovascular protection in diabetes

In conclusion, the contributions collected in this second volume highlight the urgent need for a multidimensional and anticipatory approach to cardiovascular prevention in T2D. By integrating dynamic metabolic markers, omics-based profiling, non-invasive cardiovascular phenotyping, lifestyle and nutraceutical strategies, and evidence-based pharmacological therapies, these studies move the field toward more precise and personalized risk assessment and intervention. Together, they reinforce the concept that effective cardiovascular protection in diabetes must begin early, be tailored to individual risk profiles, and leverage both technological innovation and clinical insight to reduce the global burden of diabetic cardiovascular disease.
